# Supplementary material for: Comparative Effectiveness and Safety of Fractional Laser and Fractional Radiofrequency for Atrophic Acne Scars: A Retrospective Propensity Score Analysis
Source: Life (Basel). 2025 Sep 1;15(9):1379. doi: 10.3390/life15091379 (PMC12471114; doi:10.3390/life15091379)
Supplement: Supplementary file 1 [file life-15-01379-s001.zip › Supplementary.pdf]

Supplementary Table S1. Characteristics of patients with acne scarring compared fractional laser to fractional radiofrequency treatment groups

Characteristics	Fractional laser treatment (n=254)	Fractional radiofrequency treatment (n=143)	Missing data	P-value
	**N (%) or mean ± SD**	**N (%) or mean ± SD**	**N (%)**	
**Demographic factors**				
Age (years)	33.03 ± 7.41	34.29 ± 10.67	19 (4.79)	0.174
Gender			20 (5.04)	0.076
Male	96 (40.00)	42 (30.66)		
Female	144 (60.00)	95 (69.34)		
Skin phototypes			24 (6.05)	0.734
III	56 (23.73)	37 (27.01)		
IV	148 (62.71)	84 (61.31)		
V	32 (13.56)	16 (11.68)		
**Clinical factors**				
Scar age (years)	15.91 ± 7.56	8.47 ± 3.96	60 (15.11)	**<0.001**
Severity of acne scar			26 (6.55)	**<0.001**
Almost clear - Mild	108 (11.44)	28 (5.19)		
Moderate - very severe	836 (88.56)	512 (84.81)		
Type of acne scar			26 (6.55)	**<0.001**
1	216 (22.88)	176 (32.59)		
>1	728 (77.12)	364 (67.41)		
Number of treatment sessions	3.21 ± 0.62	3.41 ± 0.49	8 (2.02%)	**0.001**

SD, standard deviation; Statistically significant P-value associations are shown in bold characters

Supplementary Table S2. Devices used in the study

Device	Key Parameters
**Fractional Laser**	
Er:YAG Laser (SP Dynamis, Fotona, Slovenia)	Pulse duration: 350 µs, Energy: 14 mJ, Pattern: 11×11 mm
CO_2 Laser (AcuPulse®, Lumenis, Israel)	Pulse duration: 950 µs, Energy: 12.5–15 mJ, Scan size: 10×10 mm
Picosecond Laser (Enlighten™, Cutera Inc., USA)	8-mm spot, Fluence: 1.0 J/cm², 2–4 passes, ~10 J/cm² microbeam
Fractional CO_2 (Ellipse Juvia, Ellipse A/S, Denmark)	Pulse width: 6 ms, Energy: 75–105 mJ, Coverage: 9.6%, 49 MTZ/cm²
Fractional CO_2 (AcuPulse®, Lumenis, Israel)	Pulse duration: 950 µs, Energy: 12.5 mJ, Coverage: 5%
1064-nm Picosecond Laser (Enlighten™, Cutera Inc., USA)	Fluence: 1 J/cm², 10 Hz, 15–20% overlap, microbeam diameter: 200 µm
**Fractional radiofrequency**	
Fractora™ (InMode Ltd., Israel)	Energy: 25–30 mJ/pin, Endpoint: mild petechiae
eMatrix™ (Syneron Medical Ltd., Israel)	Energy: 60–100 mJ/pin, Split-face application
Venus Viva™ (Venus Concept, Canada)	160 pins, Max energy: 62 mJ/pin, Voltage: 260 V, Pulse: 30 ms
INTRAcel™ (Jeisys Medical Inc., South Korea)	49 microneedles, Length: 1.5 mm, Tip: 0.3 mm, uninsulated

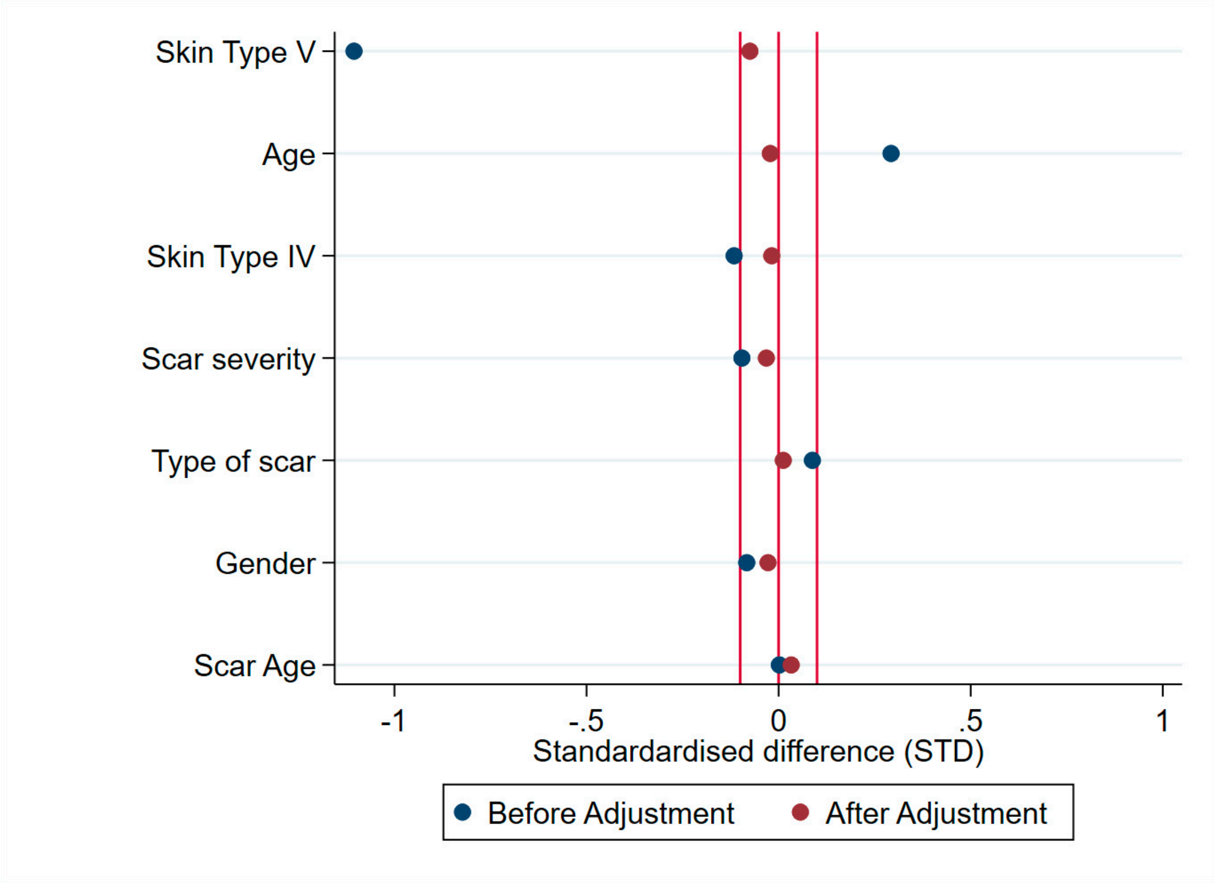

Supplementary Figure S1. Standardized differences before and after propensity score stratification adjustment

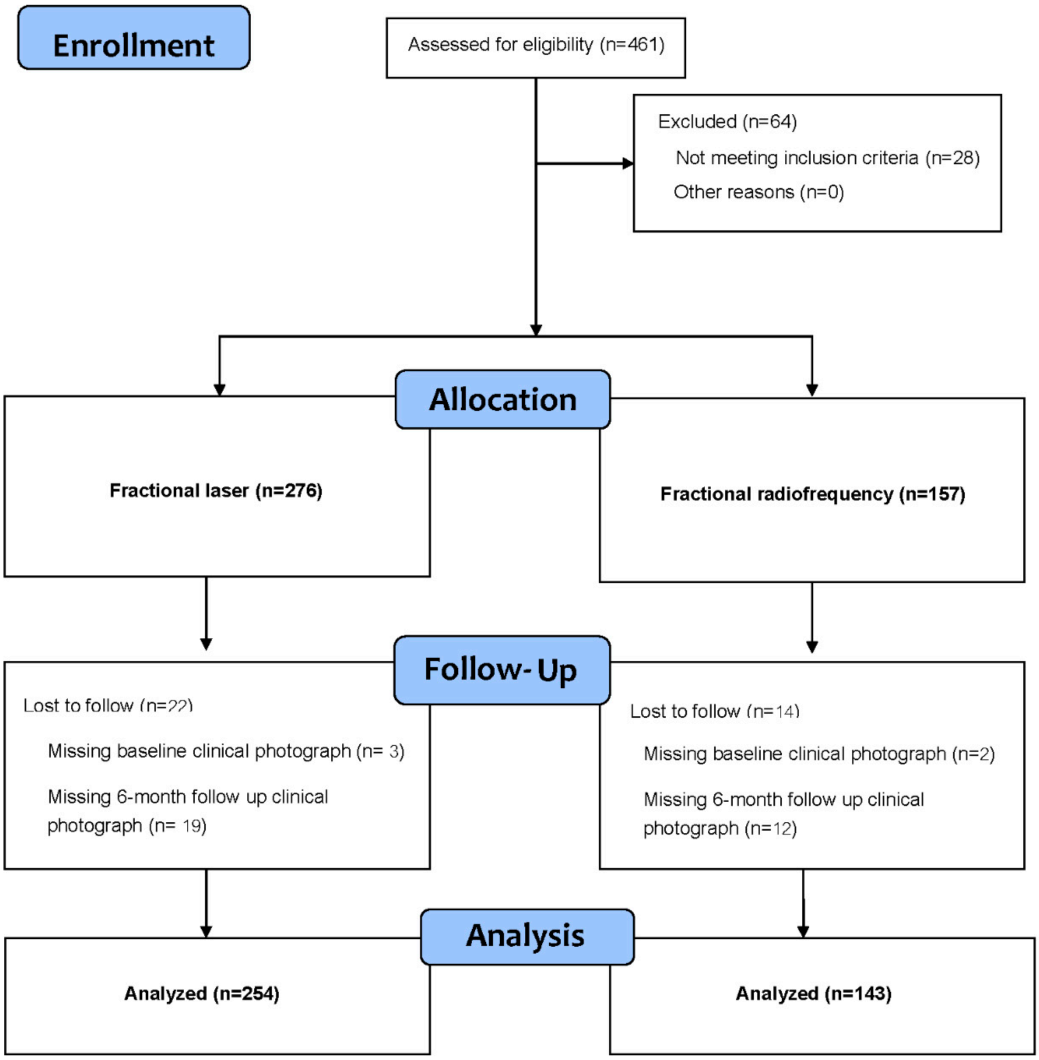

Supplementary Figure S2. Study flow diagram

Supplementary File S1. STROBE Statement—checklist of items that should be included in reports of observational studies

	Item No.	Recommendation	Page No.	Relevant text from manuscript
**Title and abstract**	1	(*a*) Indicate the study's design with a commonly used term in the title or the abstract	1	Title specifies "A Retrospective Propensity Score Analysis"
		(*b*) Provide in the abstract an informative and balanced summary of what was done and what was found	3-4	Abstract reports objectives, design, PS stratification, imputation, results, and conclusions.
**Introduction**				
Background/rationale	2	Explain the scientific background and rationale for the investigation being reported	5-6	Background on acne scars, energy-based devices, need for comparative data in Thai patients.
Objectives	3	State specific objectives, including any prespecified hypotheses	6	Objective and hypothesis: FL non-inferior to FRF in Thai patients with Fitzpatrick III–IV.
**Methods**				
Study design	4	Present key elements of study design early in the paper	6	A therapeutic efficacy research using a retrospective cohort design was conducted.
Setting	5	Describe the setting, locations, and relevant dates, including periods of recruitment, exposure, follow-up, and data collection	6	Siriraj Skin & Laser Center, 2012–2023.
Participants	6	*Cohort study*—Give the eligibility criteria, and the sources and methods of selection of participants. Describe methods of follow-up	6-7	Patients 18–60, ≥2 sessions; excluded if photographs missing.
Variables	7	Clearly define all outcomes, exposures, predictors, potential confounders, and effect modifiers. Give diagnostic criteria, if applicable	7	Outcomes: ≥25% improvement, % improvement, AEs; exposures: FL vs FRF; covariates: age, sex, scar type, severity, duration, skin type.
Data sources/ measurement	8*	For each variable of interest, give sources of data and details of methods of assessment (measurement). Describe comparability of	7	Photographic assessments by 2 dermatologists, baseline covariates from medical records.

		assessment methods if there is more than one group		
Bias	9	Describe any efforts to address potential sources of bias	7	PS stratification and balance checking with STD.
Study size	10	Explain how the study size was arrived at		Sample size estimated: ≥111 per group (total ≥222).

Quantitative variables	11	Explain how quantitative variables were handled in the analyses. If applicable, describe which groupings were chosen and why	6-7	Continuous (mean ± SD) and categorical (%) data; ≥25% threshold for improvement.
Statistical methods	12	(*a*) Describe all statistical methods, including those used to control for confounding	7-8	PS stratification, linear mixed-effects models, TOST, multiple imputation.
		(*b*) Describe any methods used to examine subgroups and interactions	7-8	Time-point analyses (1, 3, 6 months).
		(*c*) Explain how missing data were addressed	7	kNN and multiple imputation.
		(*d*) *Cohort study*—If applicable, explain how loss to follow-up was addressed	7	Excluded patients with missing photographs.
		(*e*) Describe any sensitivity analyses	8	Original vs imputed dataset; device parameters (Supplementary Tables).
**Results**				
Participants	13*	(a) Report numbers of individuals at each stage of study—eg numbers potentially eligible, examined for eligibility, confirmed eligible, included in the study, completing follow-up, and analysed	8	Total 397 patients: 254 FL, 143 FRF; exclusions explained.
		(b) Give reasons for non-participation at each stage	8	Patients were excluded if baseline or post-treatment clinical photographs were unavailable. The two-session requirement was based on previous findings indicating the clinical significance.
		(c) Consider use of a flow diagram		Flow diagram included (Supplementary Figure S2).

Descriptive data	14*	(a) Give characteristics of study participants (eg demographic, clinical, social) and information on exposures and potential confounders	8	Baseline demographics and covariates reported; PS balance shown.
		(b) Indicate number of participants with missing data for each variable of interest	8	Reported in Supplementary Table S2
		(c) *Cohort study*—Summarise follow-up time (eg, average and total amount)	8	6-month follow-ups after treatment
Outcome data	15*	*Cohort study*—Report numbers of outcome events or summary measures over time	*8-9*	≥25% improvement and % improvement at 1, 3, 6 months; Tables 2–3.
Main results	16	(*a*) Give unadjusted estimates and, if applicable, confounder-adjusted estimates and their precision (eg, 95% confidence interval). Make clear which confounders were adjusted for and why they were included	*8-9*	Unadjusted and PS-adjusted, with CIs.
		(*b*) Report category boundaries when continuous variables were categorized	*8-9*	Categorization of improvement ≥25%.
		(*c*) If relevant, consider translating estimates of relative risk into absolute risk for a meaningful time period	NA	Not relevant
Other analyses	17	Report other analyses done—eg analyses of subgroups and interactions, and sensitivity analyses	8	Sensitivity, subgroup/time comparisons reported.
**Discussion**				
Key results	18	Summarise key results with reference to study objectives	9-10	Both modalities improved scars; FL non-inferior to FRF.
Limitations	19	Discuss limitations of the study, taking into account sources of potential bias or imprecision.	12-13	Residual confounding, heterogeneity, interobserver variability, single-center.

		Discuss both direction and magnitude of any potential bias		
Interpretation	20	Give a cautious overall interpretation of results considering objectives, limitations, multiplicity of analyses, results from similar studies, and other relevant evidence	10-12	Overall discussion part was shown interpretation of results considering objectives, limitations, multiplicity of analyses, results from similar studies, and other relevant evidence
Generalisability	21	Discuss the generalisability (external validity) of the study results	13	Limited to Thai patients with skin type III–IV.
**Other information**				
Funding	22	Give the source of funding and the role of the funders for the present study and, if applicable, for the original study on which the present article is based	14	No external funding; IRB approval reported.

*Give information separately for cases and controls in case-control studies and, if applicable, for exposed and unexposed groups in cohort and cross-sectional studies.

Note: An Explanation and Elaboration article discusses each checklist item and gives methodological background and published examples of transparent reporting. The STROBE checklist is best used in conjunction with this article (freely available on the Web sites of PLoS Medicine at http://www.plosmedicine.org/, Annals of Internal Medicine at http://www.annals.org/, and Epidemiology at http://www.epidem.com/). Information on the STROBE Initiative is available at www.strobe-statement.org.
