# Supplementary figures and images for: Comparative Effectiveness and Safety of Fractional Laser and Fractional Radiofrequency for Atrophic Acne Scars: A Retrospective Propensity Score Analysis
Source: Life (Basel). 2025 Sep 1;15(9):1379. doi: 10.3390/life15091379 (PMC12471114; doi:10.3390/life15091379)

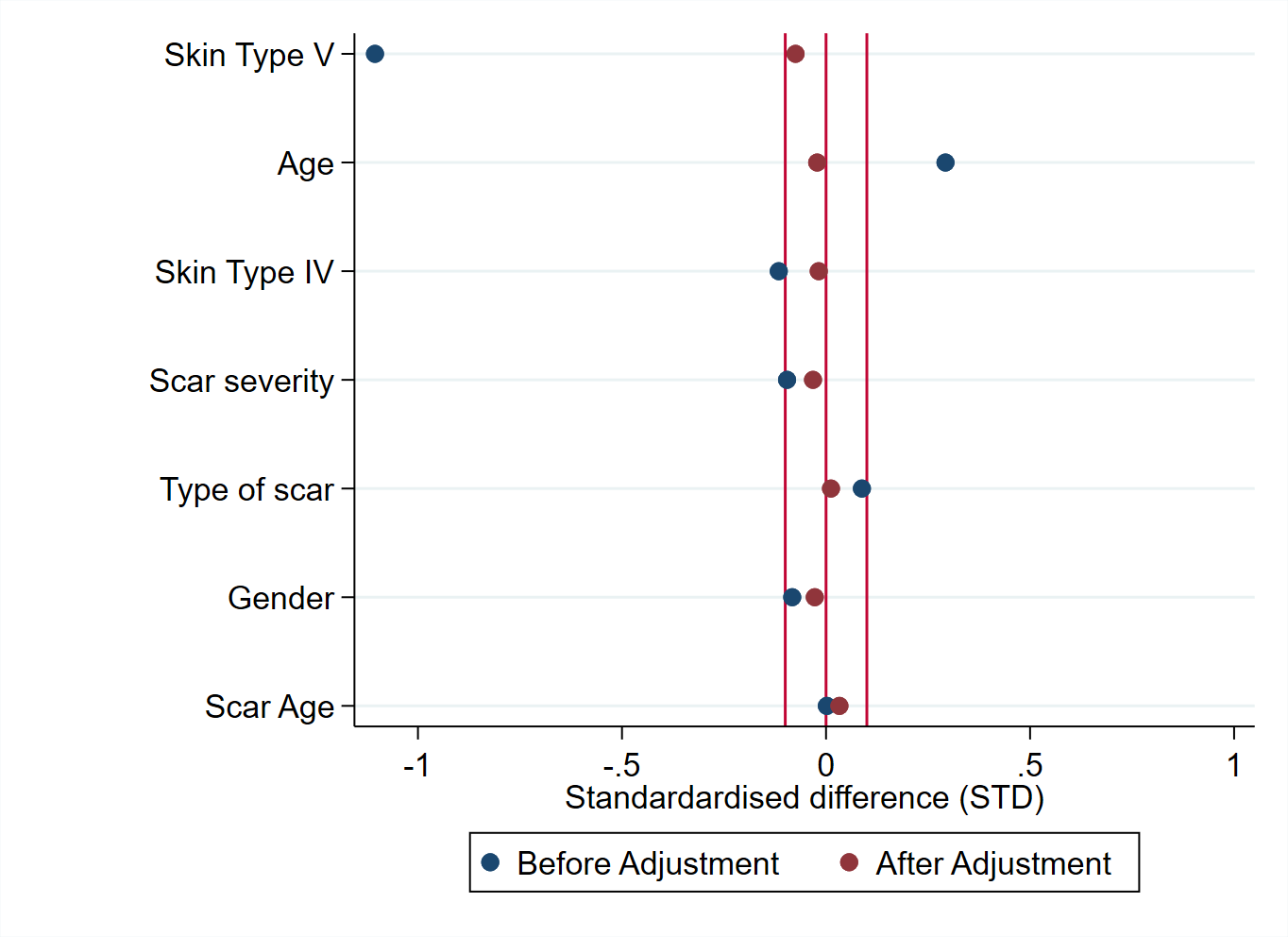

Supplement: Supplementary file 1 [file life-15-01379-s001.zip › Supplementary Figure S1.tif]

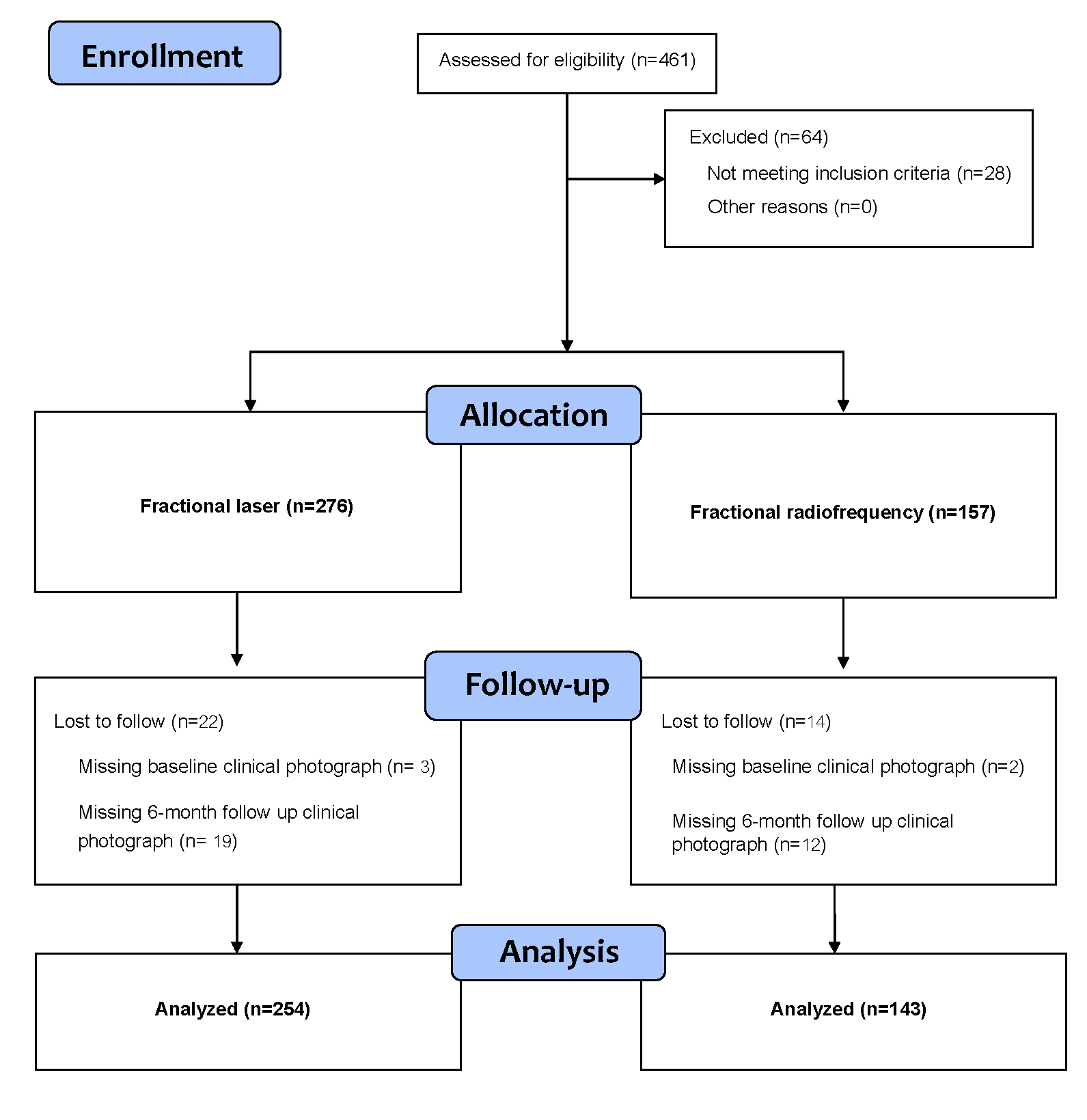

Supplement: Supplementary file 1 [file life-15-01379-s001.zip › Supplementary Figure S2.tiff]
